# Comparison of rosuvastatin and atorvastatin for lipid lowering in patients with type 2 diabetes mellitus: results from the URANUS study

**DOI:** 10.1186/1475-2840-4-7

**Published:** 2005-06-03

**Authors:** Christian Berne, Annica Siewert-Delle, the URANUS study investigators

**Affiliations:** 1 University Hospital, Uppsala, Sweden; 2 AstraZeneca Sverige AB, Sweden

## Abstract

**Objective:**

The Use of Rosuvastatin versus Atorvastatin iN type 2 diabetes mellitUS (URANUS) study compared rosuvastatin with atorvastatin for the reduction of low-density lipoprotein cholesterol (LDL-C) in patients with type 2 diabetes.

**Methods:**

After a 6-week dietary run-in, patients aged ≥ 18 years with type 2 diabetes and LDL-C ≥ 3.3 mmol/L were randomised to double-blind treatment with rosuvastatin 10 mg (n = 232) or atorvastatin 10 mg (n = 233) for 4 weeks. Doses were then titrated up to a maximum of rosuvastatin 40 mg or atorvastatin 80 mg over 12 weeks to achieve the 1998 European LDL-C goal (<3.0 mmol/L).

**Results:**

Rosuvastatin reduced LDL-C levels significantly more than atorvastatin during the fixed-dose and titration periods (p < 0.0001). Significantly more patients reached the 1998 LDL-C goal with rosuvastatin 10 mg compared with atorvastatin 10 mg at 4 weeks (81% vs 65%, p < 0.001). At 16 weeks, significantly more patients achieved their LDL-C goal with rosuvastatin compared with atorvastatin (94% vs 88%, p < 0.05) and more patients receiving rosuvastatin remained at their starting dose with reduced requirement for dose titration. At 4 weeks, 65% of rosuvastatin patients had reached their 2003 European LDL-C goal (< 2.5 mmol/L), compared with 33% of atorvastatin patients (p < 0.0001). Both treatments were similarly well tolerated with no unexpected safety concerns.

**Conclusion:**

At the start dose and following dose titration, rosuvastatin was significantly more effective than atorvastatin at reducing LDL-C and achieving European LDL-C goals in patients with type 2 diabetes.

## Introduction

The prevalence of type 2 diabetes in adults was estimated at 2.8% worldwide in 2000, and predicted to increase to 4.4% by 2030 [1]. Patients with type 2 diabetes have a risk of cardiovascular disease approximately two- to four-times greater than that in the non-diabetic population [2]. Furthermore, their prognosis is worse; in a Swedish study the 5-year mortality rate after myocardial infarction was 55% for patients with diabetes compared with 30% in patients without diabetes (p < 0.001), and the re-infarction rates were 42% and 25%, respectively (p < 0.001) [3]. More recent data reflecting the outcome of new evidence-based interventions in acute myocardial infarction demonstrate that the difference between diabetic and non-diabetic subjects is still present, showing a 1-year mortality in males of 22.3% versus 13.0% in males and 26.1% versus 14.4% in females [4]. In the US National Health and Nutrition Examination Survey (NHANES I), the age-adjusted mortality rate in diabetic patients over 9 years of follow-up was double that in non-diabetic patients, and cardiovascular disease accounted for 75% of the excess mortality in men and 57% in women [5].

The elevated cardiovascular risk in patients with type 2 diabetes is primarily attributed to the clustering of atherogenic risk factors, including dyslipidaemia, hypertension, abdominal obesity, left ventricular hypertrophy, and impaired fibrinolysis [6]. For patients with diabetes, European Diabetes Policy Group guidelines published in 1999 and European guidelines for coronary heart disease prevention published in 1998, both recommend that low-density lipoprotein cholesterol (LDL-C) levels should be <3.0 mmol/L [7,8]. More recent (2003) European guidelines on cardiovascular disease prevention also recognise type 2 diabetes as a risk factor, and recommend more stringent LDL-C reductions to<2.5 mmol/L [9]. In addition, this goal is recommended by both the American Diabetes Association and the US National Cholesterol Education Program Adult Treatment Panel III [10,11].

Statins are recognized as first-line therapy for cholesterol lowering [7,11], and have been proven to reduce cardiovascular morbidity and mortality in large outcome trials in various populations [12-17]. The benefits of statin therapy extend to patients with diabetes, as shown by subgroup analyses of patients with diabetes in several of the major statin outcome studies, including the Cholesterol And Recurrent Events (CARE) study, the Scandinavian Simvastatin Survival Study (4S), the Long-Term Intervention with Pravastatin in Ischemic Disease (LIPID) study and the Heart Protection Study [18-21]. The Collaborative Atorvastatin Diabetes Study (CARDS) recently investigated the effects of lipid lowering with statin therapy specifically in patients with type 2 diabetes [22]. The primary endpoint, time to the first occurrence of acute coronary events, coronary revascularisation or stroke, was significantly reduced by 37% in patients treated with atorvastatin 10 mg compared with placebo (p = 0.001). In addition, LDL-C levels were significantly reduced by 40% in the atorvastatin 10 mg group compared with the placebo group (p < 0.001) [22].

Despite the proven benefits of statin therapy, studies suggest that many patients with diabetes fail to achieve lipid goals in clinical practice [23,24]. Statins differ in their lipid-modifying efficacy and their ability to enable patients to achieve lipid goals [25,26]. Trials in patients with hypercholesterolaemia have shown that rosuvastatin is more effective than atorvastatin at reducing LDL-C and achieving US and/or European LDL-C goals over treatment periods ranging from 6 to 52 weeks [25-30]. The URANUS (Use of Rosuvastatin versus Atorvastatin iN type 2 diabetes mellitUS) study is a direct comparison of the effects of rosuvastatin with atorvastatin on LDL-C, other plasma lipids and LDL-C goal achievement in patients with diabetes. This study was designed to reflect the optimal treatment of diabetic dyslipidaemia in the clinical setting, in that statin dose was titrated upwards from the recommended start dose to that required to enable patients to achieve LDL-C goal.

## Methods

### Patients

Patients (male or female) aged 18 years or more were eligible for the study if they had a history of type 2 diabetes for at least 3 months; were being treated with diet, oral antidiabetic medication, insulin or a combination of these treatments; and had fasting LDL-C of ≥ 3.3 mmol/L and triglycerides (TG) of<6.0 mmol/L at enrolment. Exclusion criteria included: type 1 diabetes; uncontrolled type 2 diabetes; uncontrolled hypothyroidism or hypertension; nephrotic syndrome or severe renal failure; active liver disease or hepatic dysfunction; active arterial disease (e.g., unstable angina, myocardial infarction, transient ischaemic attack, cerebrovascular accident, coronary artery bypass grafting or percutaneous transluminal coronary angioplasty within 3 months before beginning the study); serum creatinine kinase (CK) levels >3 × the upper limit of normal (ULN); body mass index >35 kg/m^2^; and known hypersensitivity to statins. All patients gave written informed consent, and the study was conducted in accordance with the Declaration of Helsinki.

### Study design

**Figure 1:**
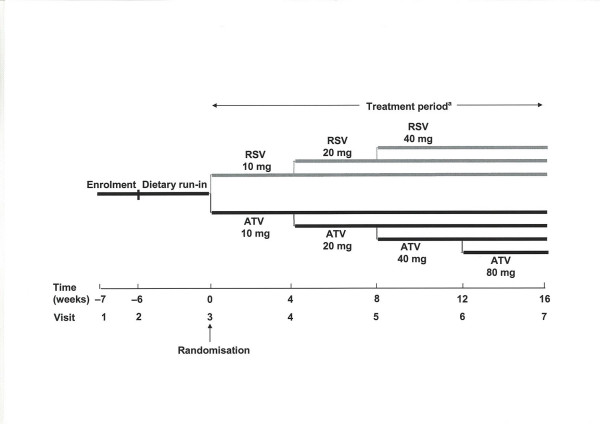
**Study design**. ^a^In patients who had not reached the European goal of LDL-C<3.0 mmol/L after 4 weeks, the statin dose was doubled at each visit, up to a maximum of RSV 40 mg and ATV 80 mg **RSV**: Rosuvastatin, **ATV**: Atorvastatin, **LDL-C**: Low-density lipoprotein cholesterol

The trial was a randomised, double-blind, parallel-group study (4522SE/0001) conducted in 51 centres in Sweden. The study design is summarised in figure 1. Patients meeting the inclusion and exclusion criteria at enrolment entered a 6-week dietary run-in period and all lipid-lowering therapy was withdrawn at least 14 days before the end of this period. Patients with fasting LDL-C ≥ 3.3 mmol/L were then randomised to a starting dose of either rosuvastatin 10 mg or atorvastatin 10 mg for 4 weeks. This was followed by a 12-week period of dose titration, making a total of 16 weeks of treatment. Patients who had not reached the 1998 European guideline goal of LDL-C<3.0 mmol/L [7] after 4 weeks were titrated up by doubling the statin dose (rosuvastatin 20 mg or atorvastatin 20 mg). Further dose titrations (to rosuvastatin 40 mg, or atorvastatin 40 mg or 80 mg) were performed at 8 weeks and 12 weeks for patients who were still not at their LDL-C goal. Patients whose LDL-C level was below the goal at 4 weeks continued on the initial dose of study medication; if their LDL-C level exceeded the goal at subsequent visits, study medication was up-titrated.

Concomitant treatment with erythromycin, azole antimycotic agents, vitamin K antagonists, immunosuppressive agents, glitazones or systemic steroids was not permitted during the study. If insulin treatment became necessary, or if the patient took lipid-lowering medication (other than study medication), the patient was discontinued from the trial.

### Assessments

#### Efficacy

The primary endpoint was the percentage change in LDL-C from baseline (randomisation) to 16 weeks. Secondary endpoints included: percentage change in LDL-C from baseline to 4 weeks; percentage of patients achieving the 1998 European LDL-C goal at 4 and 16 weeks; percentage change in total cholesterol (TC), TG, high-density lipoprotein cholesterol (HDL-C), the LDL-C/HDL-C ratio, the non-HDL-C/HDL-C ratio, the TC/HDL-C ratio, apolipoprotein (apo) B, apo A-I and the apo B/apo A-I ratio from baseline to 4 and 16 weeks; and the number of titration steps at 16 weeks. A tertiary endpoint was the difference in overnight urinary albumin excretion (UAE) from baseline to 16 weeks.

All patients were instructed to fast for 8 hours prior to giving blood samples. LDL-C levels were measured using a direct method with enzymatic colorimetry (Genzyme Diagnostics, Genzyme Corporation, Cambridge, MA, USA). All analyses were conducted at a central laboratory.

#### Safety

Adverse events spontaneously reported by the patients, elicited in response to an open question or revealed by observation, were recorded at each visit. Laboratory safety variables included: blood haemoglobin, platelet count, leucocyte count, serum aspartate aminotransferase (ASAT), serum alanine aminotransferase (ALAT), serum alkaline phosphatase, serum bilirubin, CK, serum creatinine and glycated haemoglobin (HbA_1c_). All analyses were performed at a central laboratory.

### Statistical methods

In order to have a 90% chance of detecting a difference between the two treatment arms of 6% in the primary endpoint (percentage change in LDL-C from baseline to 16 weeks), 212 patients per arm were required to complete the study. The primary efficacy endpoint was determined using analysis of covariance with change in LDL-C as response variable, treatment and centre as factors, and baseline LDL-C as covariate. Percentage change in other lipid variables from baseline to 4 weeks and 16 weeks, and percentage change in UAE, were analysed in the same way as the primary endpoint. The proportion of patients reaching LDL-C goal was analysed using a Mantel-Haenszel test stratified by centre. All tests were two-sided with a significance level of 5%. In addition, 95% confidence intervals were calculated for the treatment differences in all efficacy variables except the percentage of patients reaching LDL-C goal.

All efficacy variables were analysed in the intention-to-treat population (observed data). Analysis of the primary endpoint was also carried out using the last observation carried forward approach. All enrolled patients were evaluated for safety.

## Results

### Demographics

**Figure 2:**
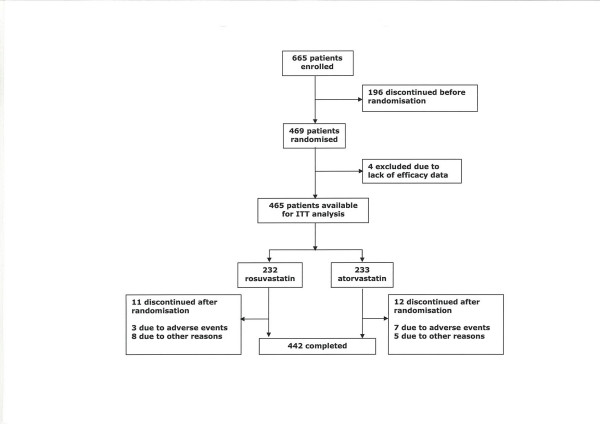
Study populations

A total of 469 patients were randomised, and efficacy data were obtained from 465 patients, 232 in the rosuvastatin group and 233 in the atorvastatin group (figure 2). The two groups were well matched at baseline, and demographic details are shown in table 1. Previous statin treatment was received by 31 patients (13%) in the rosuvastatin group and 39 patients (17%) in the atorvastatin group. Eleven patients in the rosuvastatin group and 12 in the atorvastatin group discontinued during the randomised treatment period (figure 2).

**Table 1:** Patient demographics of ITT population

	**Rosuvastatin (n = 232)**	**Atorvastatin (n = 233)**
Gender, male/female (%)	128/104 (55.2/44.8)	136/97 (58.4/41.6)
Race, white (%)	229 (98.7)	229 (98.3)
Mean age, years (SD)	63.5 (8.8)	65.0 (8.6)
Mean weight, kg (SD)	84.8 (14.3)	82.5 (13.5)
Mean BMI, kg/m^2 ^(SD)	29.0 (3.6)	28.4 (3.6)
Mean baseline LDL-C, mmol/L (SD)	4.6 (0.85)	4.6 (0.82)^a^
Mean baseline HDL-C, mmol/L (SD)	1.2 (0.27)	1.2 (0.27)^a^
Mean baseline TG, mmol/L (SD)	2.0 (1.0)	2.0 (0.93)^a^

**ITT: **Intention to treat, **SD: **Standard deviation, **BMI: **Body mass index, **LDL-C: **Low-density lipoprotein cholesterol, **HDL-C: **High-density lipoprotein cholesterol, **TG: **Triglycerides

^a^n = 232

### Efficacy

**Figure 3:**
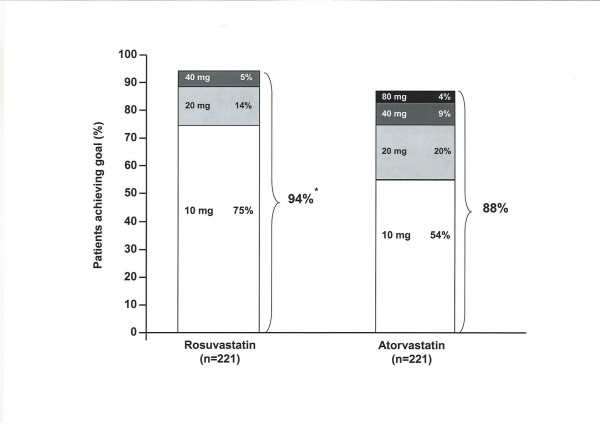
**Cumulative percentage of patients to 1998 European LDL-C goal of<3.0 mmol/L [7] by dose at 16 weeks**. *p < 0.05 rosuvastatin 10–40 mg vs atorvastatin 10–80 mg **RSV**: Rosuvastatin, **ATV**: Atorvastatin, **LDL-C**: Low-density lipoprotein cholesterol

At the end of the titration-to-goal period, rosuvastatin was significantly more effective than atorvastatin on the primary efficacy measure, reducing LDL-C by 52% compared with 46% in the atorvastatin group (p < 0.0001) (table 2). In line with its greater efficacy for LDL-C reduction, significantly more rosuvastatin-treated patients reached the 1998 European LDL-C goal after 16 weeks than atorvastatin-treated patients (94% vs 88%, p < 0.05). Furthermore, more patients achieved the goal on the starting dose of rosuvastatin than atorvastatin (75% vs 54%) (figure 3). The greater ability of rosuvastatin to lower LDL-C was also reflected by the number of dose titrations required by each treatment group; a total of 75 titration steps were required by rosuvastatin-treated patients compared with 155 titrations in the atorvastatin group.

**Table 2:** Percentage change from baseline in lipid variables at 16 weeks (ITT population). Doses were titrated from week 4 to week 16 in patients who had not reached the 1998 European LDL-C goal (< 3.0 mmol/L)

**Variable**	**Least-squares mean percentage change from baseline to 16 weeks**		**Difference (95% CI)**	**p-value**
			
	**Rosuvastatin 10–40 mg (n = 221)**	**Atorvastatin 10–80 mg (n = 220)**		
LDL-C	-52.3	-45.5	-6.7 (-8.8, -4.7)	< 0.0001
TC	-35.4	-31.3	-4.1 (-5.8, -2.4)	< 0.0001
HDL-C	5.3	4.0	1.3 (-1.3, 3.8)	NS
TG	-21.2	-21.1	-0.1 (-5.6, 5.3)	NS
Non-HDL-C	-45.0	-39.6	-5.5 (-7.4, -3.5)	< 0.0001
LDL-C/HDL-C ratio	-54.1	-47.0	-7.1 (-9.3, -4.9)	< 0.0001
Non-HDL-C/HDL-C ratio	-47.1	-40.9	-6.2 (-8.6, -3.9)	< 0.0001
TC/HDL-C ratio	-38.0	-33.1	-5.0 (-6.9, -3.0)	< 0.0001
Apo B	-45.2	-40.1	-5.1 (-7.2, -3.1)	< 0.0001
Apo A-I	2.6	-0.2	2.8 (1.0, 4.6)	0.0024
Apo B/apo A-I ratio	-46.3	-39.6	-6.7 (-8.9, -4.6)	< 0.0001

**ITT: **Intention to treat, **CI: **Confidence interval, **LDL-C: **Low-density lipoprotein cholesterol, **TC: **Total cholesterol, **HDL-C: **High-density lipoprotein cholesterol, **TG: **Triglycerides, **Apo: **Apolipoprotein, **NS: **Not statistically significant

**Figure 4:**
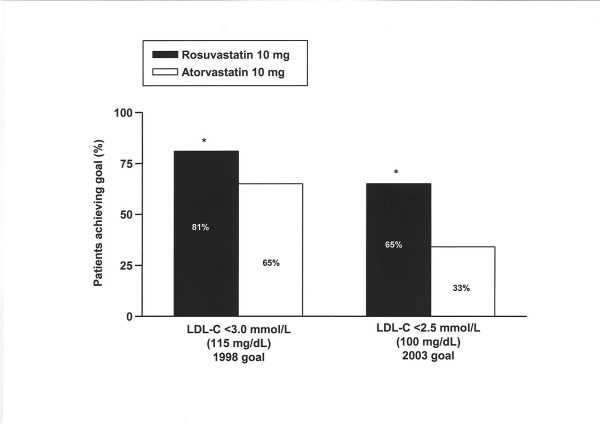
**Percentage of patients to 1998 and 2003 European LDL-C goals [7,9] at 4 weeks**. *p < 0.001 vs atorvastatin **LDL-C**: Low-density lipoprotein cholesterol

During the 4-week fixed-dose period, significantly more patients on rosuvastatin 10 mg had reached the 1998 European LDL-C goal compared with patients on atorvastatin 10 mg (figure 4). When data from the fixed-dose period were re-analysed to the more stringent 2003 European LDL-C goal of <2.5 mmol/L, 65% of patients receiving rosuvastatin 10 mg achieved goal compared with 33% of patients receiving atorvastatin 10 mg (p < 0.001; figure 4).

Rosuvastatin also reduced TC, non-HDL-C, LDL-C/HDL-C ratio, non-HDL-C/HDL-C ratio, and TC/HDL-C ratio significantly (p < 0.0001) more than atorvastatin after 4 weeks of treatment (table 3). Both treatments increased HDL-C and decreased TG from baseline to 4 weeks, but there were no statistically significant differences between the groups (table 3). In addition, rosuvastatin significantly reduced levels of apo B and the apo B/apo A-I ratio, and increased apo A-I levels compared with atorvastatin (p ≤ 0.05) (table 3). Similar significant effects on TC, non-HDL-C, apolipoproteins and lipid ratios were observed at 16 weeks (table 2).

**Table 3:** Percentage change from baseline in lipid variables at 4 weeks (ITT population)

**Variable**	**Least-squares mean percentage change from baseline to 4 weeks**		**Difference (95% CI)**	**p-value**
			
	**Rosuvastatin 10 mg (n = 232)**	**Atorvastatin 10 mg (n = 231)**		
LDL-C	-47.6	-38.5^a^	-9.1 (-11.4, -6.7)	< 0.0001
TC	-33.6	-27.9	-5.7 (-7.4, -4.0)	< 0.0001
HDL-C	4.4	2.6	1.8 (-0.5, 4.0)	NS
TG	-19.2	-15.5	-3.7 (-9.5, 2.2)	NS
Non-HDL-C	-42.6	-35.0	-7.6 (-9.6, -5.7)	< 0.0001
LDL-C/HDL-C ratio	-49.3	-39.5	-9.8 (-12.2, -7.4)	< 0.0001
Non-HDL-C/HDL-C ratio	-44.4	-35.9	-8.5 (-10.8, -6.2)	< 0.0001
TC/HDL-C ratio	-35.8	-29.0	-6.7 (-8.7, -4.8)	< 0.0001
Apo B	-42.9	-35.3	-7.6 (-9.7, -5.6)	< 0.0001
Apo A-I	2.6	0.8	1.8 (0, 3.5)	0.05
Apo B/apo A-I ratio	-43.9	-35.4	-8.5 (-10.6, -6.4)	< 0.0001

**ITT: **Intention to treat, **CI: **Confidence interval, **LDL-C: **Low-density lipoprotein cholesterol, **TC: **Total cholesterol, **HDL-C: **High-density lipoprotein cholesterol, **TG: **Triglycerides, **Apo: **Apolipoprotein, **NS: **Not statistically significant

^a^n = 232

There was no statistically significant difference in UAE rate from baseline to study end, or between the treatment groups, including those patients with baseline microalbuminuria (UAE >20 μg/min).

### Safety

Both treatments were well tolerated, with overall incidences of adverse events being similar between the treatment groups (51% with rosuvastatin, 53% with atorvastatin). A total of 10 patients experienced serious adverse events (two in the rosuvastatin group, eight in the atorvastatin group), none of which were considered by the investigator to be related to study treatment. Ten patients discontinued because of adverse events, three in the rosuvastatin group and seven in the atorvastatin group. There were no cases of myopathy. Myalgia was reported by 3.4% of the patients in the study; none of the cases were associated with a clinically important elevation in CK (>5 × ULN). Indeed, there were no clinically important elevations in CK in either group throughout the study and changes in CK were not related to dose of study medication or duration of treatment. The most frequent adverse events overall were nasopharyngitis, myalgia, and inadequately controlled diabetes mellitus (table 4). There were no clinically relevant changes in ALAT or ASAT (>3 × ULN).

**Table 4:** Adverse events occurring in ≥ 3% of patients in any treatment group

	**Number (%) of patients with adverse event**	
**Adverse event**	**Rosuvastatin (n = 233)**	**Atorvastatin (n = 236)**

Nasopharyngitis	23 (9.9)	19 (8.1)
Myalgia	13 (5.6)	7 (3.0)
Inadequately controlled diabetes mellitus	14 (6.0)	11 (4.6)
Constipation	9 (3.9)	6 (2.5)
Headache	6 (2.6)	7 (3.0)
Urinary tract infection	9 (3.9)	2 (0.9)
Arthralgia	1 (0.4)	9 (3.8)

## Discussion

Rosuvastatin provided significantly greater LDL-C reductions than atorvastatin, both at the initial dose of 10 mg and also when titrated over the dose range of 10–40 mg for rosuvastatin and 10–80 mg for atorvastatin. In addition, a significantly higher percentage of patients treated with rosuvastatin achieved the 1998 European LDL-C goal (<3.0 mmol/L), both with the starting dose of 10 mg and after the period of dose titration. These results are consistent with the findings of studies in patients with hypercholesterolaemia that compared rosuvastatin with atorvastatin over 6 weeks [25], 8 weeks [31], 12 weeks [27,28], and 52-week dose titration [28].

Patients with type 2 diabetes commonly have a highly atherogenic lipid profile including elevated LDL-C, increased TG and low HDL-C, which is associated with a high risk of developing cardiovascular disease [32,33]. Statins are recognized as first-line therapy for cholesterol lowering, and their benefits have been shown to extend to patients with diabetes [18-22]. The present study was designed to reflect the treatment of diabetic dyslipidaemia in the clinical setting, in that statin treatment of patients was titrated upwards from the recommended starting dose to that required to achieve the 1998 European target of LDL-C <3.0 mmol/L. The population of the present study was compared with patients with type 2 diabetes in the Swedish National Diabetes Registry and was found to be consistent in terms of baseline characteristics such as age, HbA_1c_, body mass index, percentage of smokers, blood pressure, and antidiabetic medication [34].

New European guidelines published in 2003 recommend a more stringent target (LDL-C <2.5 mmol/L) [9] than that used when the present study was planned. Further analysis of the 4-week (fixed-dose) LDL-C data indicated that rosuvastatin 10 mg treated significantly more patients to the new 2003 European goal of <2.5 mmol/L than atorvastatin 10 mg. As expected, the absolute percentages of patients achieving the more stringent 2003 goal were lower than the absolute percentages achieving the 1998 goal at 4 weeks, but the greater efficacy of rosuvastatin 10 mg compared with atorvastatin 10 mg remained the same. As more clinical trial evidence becomes available regarding the positive effects of intensive lipid lowering among patients with diabetes, it is likely that even more stringent LDL-C goals will be recommended. Indeed, National Cholesterol Education Program Adult Treatment Panel III recommendations were recently reviewed and a target of LDL-C <70 mg/dL (1.8 mmol/L) was suggested as a therapeutic option for individuals considered to be at very high risk including those with both type 2 diabetes and established cardiovascular disease [35].

The availability of an agent that enables a large number of patients to achieve LDL-C goal at the starting dose is important given that many patients receiving lipid-lowering therapy fail to existing attain lipid targets due to a lack of dose titration and the use of less effective agents [36,37]. A recent observational study, designed to reflect dyslipidaemia treatment in the clinical setting, evaluated the number of hyperlipidaemic patients with coronary heart disease or diabetes who achieved LDL-C goal with their initial statin dose and whether patients were dose titrated [38]. Less than half (48%) achieved LDL-C <2.6 mmol/L with their initial dose and, of those who did not achieve goal, only 45% had their dose titrated. Dose titration increases costs and the need for follow-up, which, while necessary, can be time-consuming and inconvenient. The ability of rosuvastatin to enable greater proportions of patients to achieve LDL-C goal, with reduced requirement for dose titration is highly advantageous.

The benefits of reaching treatment goals have been demonstrated in the Steno-2 study [39], in which patients with type 2 diabetes were randomised to receive conventional treatment or intensive multifactorial intervention to strict treatment goals (including TC<4.5 mmol/L). LDL-C levels were reduced by 47% in those receiving intensive therapy, and the risk of both cardiovascular and microvascular events was reduced by approximately 50% compared with conventional treatment [39].

Rosuvastatin was also more effective than atorvastatin in reducing a range of other lipid variables, including TC, non-HDL-C, LDL-C/HDL-C ratio, non-HDL-C/HDL-C ratio, and TC/HDL-C ratio. Reductions in TC and/or TC/HDL-C are particularly relevant given that the Systemic Coronary Risk Evaluation (SCORE) system advocated in the new European guidelines uses these variables to estimate total risk [9].

In the present study, both treatments produced similar increases in HDL-C, which were lower than those observed previously. In the Measuring Effective Reductions in Cholesterol Using Rosuvastatin therapY (MERCURY I) study involving 3,161 patients with hypercholesterolaemia, 8 weeks' treatment with rosuvastatin 10 mg increased HDL-C by 9.2% and this was significantly greater than atorvastatin 10 mg (6.8%) and atorvastatin 20 mg (5.7%) (p < 0.01) [31]. In the current study, average baseline HDL-C values were higher than would be expected in the diabetic population, which could partly explain the relatively small increases in HDL-C compared with other studies [31,40].

In the present study, the main apolipoprotein in HDL-C, apo A-I, was significantly increased by rosuvastatin compared with atorvastatin (p < 0.05). In addition, significant reductions were also observed in apo B and apo B/apo A-I (p < 0.0001). Changes in apolipoprotein levels may have important implications in the reduction of cardiovascular risk, since results from the Apolipoprotein-related Mortality Risk (AMORIS) study indicate that apo B, apo A-I and apo B/apo A-I are powerful predictors of cardiac events [41]. Taken together with the other changes to lipid variables, the findings of the present study indicate that a less atherogenic lipid profile was achieved with rosuvastatin.

Treatment of diabetic dyslipidaemia may also reduce the incidence of microvascular disease including nephropathy [33]. Statins have been shown to have beneficial effects in diabetic nephropathy by reducing the rate of UAE [42,43]. In the present study, statin treatment did not significantly alter the rate of UAE; however, this may reflect the fact that the treatment period was relatively short. Previously, a reduced UAE rate with statin therapy has been observed after at least 6 months' treatment [42,43].

Both treatments were similarly well tolerated, with no unexpected safety concerns, and tolerability was similar to that previously observed in non-diabetic patient populations [40,44].

In conclusion, rosuvastatin was significantly more effective at reducing LDL-C and achieving European LDL-C goals both during the fixed-dose period and following dose titration than atorvastatin in patients with type 2 diabetes.

## Competing interests

This study was supported by AstraZeneca, Södertälje, Sweden, which provided study expenses and covered the article processing charge. Annica Siewert-Delle is an employee of AstraZeneca.

## Authors' contributions

Christian Berne and Annica Siewert-Delle participated in the design and coordination of the study and prepared the manuscript. Both authors read and approved the final manuscript.
